# Knockdown of Musashi RNA Binding Proteins Decreases Radioresistance but Enhances Cell Motility and Invasion in Triple-Negative Breast Cancer

**DOI:** 10.3390/ijms21062169

**Published:** 2020-03-21

**Authors:** Fabian M. Troschel, Annemarie Minte, Yahia Mahmoud Ismail, Amr Kamal, Mahmoud Salah Abdullah, Sarah Hamdy Ahmed, Marie Deffner, Björn Kemper, Ludwig Kiesel, Hans Theodor Eich, Sherif Abdelaziz Ibrahim, Martin Götte, Burkhard Greve

**Affiliations:** 1 Department of Radiation Oncology, University Hospital Münster, 48149 Münster, Germany; fabian.troschel@web.de (F.M.T.); annemarie.kohl@gmx.de (A.M.); m_kuhl13@uni-muenster.de (M.D.); hans.eich@ukmuenster.de (H.T.E.); 2 Department of Gynecology and Obstetrics, University Hospital Münster, 48149 Münster, Germany; Ludwig.Kiesel@ukmuenster.de; 3 Medical Oncology Department, National Cancer Institute, Cairo University, 11796 Cairo, Egypt; yahia.ismail@nci.cu.edu.eg; 4 Surgical Oncology Department, National Cancer Institute, Cairo University, 11796 Cairo, Egypt; amrnci@gmail.com; 5 Biotechnology/Biomolecular Chemistry program, Chemistry Department, Faculty of Science, Cairo University, 12613 Giza, Egypt; mahmoudrete@icloud.com (M.S.A.); sarahhamdy622@yahoo.com (S.H.A.); 6 Biomedical Technology Center, Medical Faculty, University of Münster, 48149 Münster, Germany; bkemper@uni-muenster.de; 7 Zoology Department, Faculty of Science, Cairo University, 12613 Giza, Egypt; isherif@sci.cu.edu.eg

**Keywords:** Musashi RNA-binding proteins, breast cancer stem cells, Notch, apoptosis, proliferation, radiotherapy, EGFR, LIFR, migration, invasiveness

## Abstract

The therapeutic potential of Musashi (MSI) RNA-binding proteins, important stemness-associated gene expression regulators, remains insufficiently understood in breast cancer. This study identifies the interplay between MSI protein expression, stem cell characteristics, radioresistance, cell invasiveness and migration. MSI-1, MSI-2 and Notch pathway elements were investigated via quantitative polymerase chain reaction (qPCR) in 19 triple-negative breast cancer samples. Measurements were repeated in MDA-MB-231 cells after MSI-1 and -2 siRNA-mediated double knockdown, with further experiments performed after *MSI* silencing. Flow cytometry helped quantify expression of CD44 and leukemia inhibitory factor receptor (LIFR), changes in apoptosis and cell cycle progression. Proliferation and irradiation-induced effects were assessed using colony formation assays. Radiation-related proteins were investigated via Western blots. Finally, cell invasion assays and digital holographic microscopy for cell migration were performed. MSI proteins showed strong correlations with Notch pathway elements. *MSI* knockdown resulted in reduction of stem cell marker expression, cell cycle progression and proliferation, while increasing apoptosis. Cells were radiosensitized as radioresistance-conferring proteins were downregulated. However, *MSI*-silencing-mediated LIFR downregulation resulted in enhanced cell invasion and migration. We conclude that, while *MSI* knockdown results in several therapeutically desirable consequences, enhanced invasion and migration need to be counteracted before knockdown advantages can be fully exploited.

## 1. Introduction

Breast cancer stem cells (BCSCs) are a subpopulation of highly tumorigenic, radio- and chemo-resistant tumor cells [1]. They are known to play key roles in cancer initiation, progression and metastasis [2]. Given their prominent role in malignancies, novel research aims to identify and target BCSCs [3]. Several overlapping but not identical markers have been identified to quantify BCSCs, including CD44, aldehyde dehydrogenase (ALDH) and multidrug resistance (MDR)-efflux systems [4,5]. Numerous studies have been published in search for pathways to downregulate BCSC characteristics [6,7].

Musashi RNA-binding proteins (MSIs) are small intracellular proteins that have been discovered to play a key role in the post-transcriptional regulation of gene expression [8]. Two different proteins have been identified, Musashi-1 (MSI-1) and Musashi-2 (MSI-2) [9]. These two molecules show a more than 90% homology in the RNA-binding domain [9,10] and thus share most functions in a complementary way [11]. While their influence has been described to be wide-ranging, research focuses primarily on their role in cancer initiation and progression [12]. Most prominently, they have been described as crucial cancer stem cell regulators [9] in multiple entities, including in ovarian [13] and endometrial cancer [14,15]. In breast cancer, some controversy remains. Some studies identify both MSI-1 and MSI-2 as potential therapeutic targets: MSI-1 has been described to enhance BCSC characteristics through proteasome subunit expression regulation [16], and anti-tumor effects subsequent to MSI-2 targeting have been shown in breast cancer [17]. Another investigation agrees and hypothesizes that MSI-1 and MSI-2 may be suitable targets for therapy [18]. However, a new study makes a compelling case that MSI-2 is responsible for estrogen receptor 1 expression and may be associated with a good prognosis [19], suggesting that targeting MSI-2 may be unwise. Thus, the therapeutic potential of *MSI* knockdown remains largely unclear.

Radiation research has so far identified MSI-1 as a marker of radioresistance in two tumor entities only, in glioblastoma [20,21] and in colon cancer [22]. There are no data on other tumor entities, necessitating further study. 

Given an increasing drive to identify pathway-driven mechanisms that may aid breast cancer therapy, we set out to understand the role of MSI proteins in this setting. We specifically aimed to examine the interplay between MSI protein expression, stem cell characteristics, radioresistance, and cell invasiveness and migration.

## 2. Results

### 2.1. MSI Protein mRNAs Show Strong Correlations with Each Other and Notch Pathway Elements in Triple-Negative Breast Cancer Samples

To investigate *MSI* expression in breast cancer, tissue samples were collected from 19 triple-negative breast cancer (TNBC) patients. Mean age was 52 years (range 34–63) with a majority of the women in postmenopausal state. Most tumors were assessed as T2 (47%) and grade II (89%). Lymphovascular invasion was present in less than half of the cases. Patient data are summarized in Table 1.

**Figure 1:**
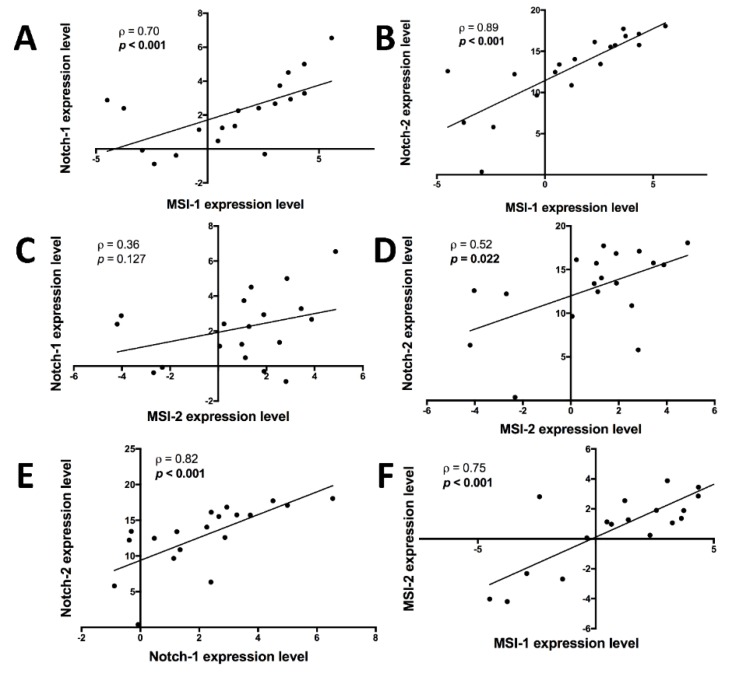
Correlations between mRNAs of *Notch-1*, *Notch-2*, *Musashi-1* (*MSI-1*) and *Musashi-2* (*MSI-2*), as measured by quantitative polymerase chain reaction (qPCR) in 19 triple-negative breast cancer patient samples. After normalization to the housekeeping gene, respective gene expression was evaluated using the 2^−∆∆Ct^ method with data then presented as log2-transformed fold change. Spearman’s ⍴ and respective *p* value (in bold if *p* < 0.05) are given for each correlation. **A**: *MSI-1* expression is positively correlated with *Notch-1* expression. **B**: *MSI-1* expression is positively correlated with *Notch-2* expression. **C**: *MSI-2* expression is not significantly correlated with *Notch-1* expression, though trending towards a positive correlation. **D**: *MSI-2* expression is positively correlated with *Notch-2* expression. **E**: *Notch-1* expression is positively correlated with *Notch-2* expression. **F**: *MSI-1* expression is positively correlated with *MSI-2* expression.

In these primary samples, mRNA analyses of *MSI-1* and *MSI-2* as well as Notch pathway elements *Notch-1* and *Notch-2* revealed significant correlations: *MSI-1* was positively correlated with *Notch-1* (Figure 1A) and *Notch-2* (Figure 1B) while *MSI-2* showed a non-significant positive correlation trend with *Notch-1* (Figure 1C) and a positive correlation with *Notch-2* (Figure 1D). Unsurprisingly, *Notch-1* and *Notch-2* were also correlated (Figure 1E). Finally, *MSI-1* and *MSI-2* were strongly correlated with each other (Figure 1F). 

When comparing the 19 TNBC tissues against 5 healthy samples obtained during reduction mammoplasty, both *Notch-1* (*p* < 0.05) and *Notch-2* (*p* < 0.01) levels were elevated in the cancerous tissue, though no changes were seen in *MSI-1* and *MSI-2* (Supplementary Figure S1).

### 2.2. MSI-1 and MSI-2 Small Interfering RNA (siRNA) Transfection Results in MSI-1 and MSI-2 Knockdown

Given homology between MSI-1 and MSI-2 [9,10] and strong expression correlations in patient samples as demonstrated above, our experimental interest was to target both MSI proteins to prevent potential compensatory effects. As success of knockdown was vital for the validity of the study, we performed qPCR analyses to evaluate knockdown success for both *MSI-1* and *MSI-2*. Expression of both mRNAs was suppressed by at least 80% (Supplementary Figure S2). Additional Western blot analysis of MSI-2 confirmed these results (Supplementary Figure S3).

### 2.3. Knockdown of MSI-1 and MSI-2 Suppresses the Notch Pathway

Previous results indicated that both MSI-1 and MSI-2 are likely to enhance Notch signaling pathway activity via targeting and downregulating a pathway inhibitor, mammalian numb (mnumb) [23]. Based on these findings and based on the strong correlation between MSI and Notch pathway elements shown in primary breast tissues above, we performed qPCR analyses to evaluate *MSI* knockdown effects on the Notch pathway in triple-negative MDA-MB-231 cells. 

**Figure 2:**
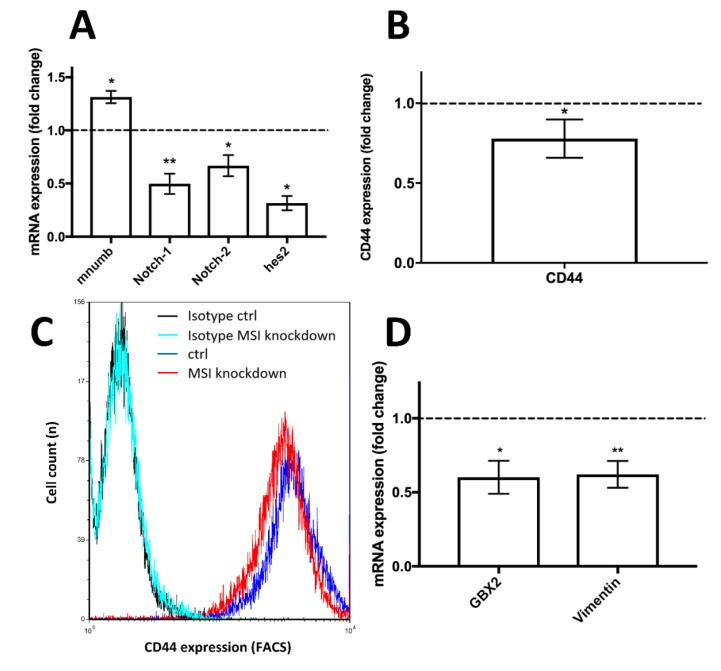
Influence of *Musashi* (*MSI*) RNA-binding protein knockdown on the Notch pathway and additional stem cell markers in triple-negative MDA-MB-231 cells. **A**: Upregulation of the Notch pathway repressor *mammalian numb* (*mnumb*) and downregulation of Notch pathway elements subsequent to *MSI-1* and *-2* knockdown compared to controls, as measured by quantitative polymerase chain reaction (qPCR). **B**: Downregulation of stem cell marker CD44 after *MSI* knockdown compared to controls, as determined by flow cytometry. Representative measurement shown in **C** (on a logarithmic x scale), including respective isotypes (unspecific antibodies of the same subclass that show low fluorescence intensity and no discernible difference between samples, thus indicating that changes are due to specific antibody binding). **D**: Downregulation of the stem cell markers *GBX2* and *vimentin* subsequent to *MSI* knockdown as determined by qPCR. Cells were transfected with a control siRNA and *MSI-1* and *MSI-2* siRNA, respectively, as detailed in the Methods section (at least *n* = 3, * *p* < 0.05, ** *p* < 0.01, error bars indicate standard error of the mean (s.e.m.)).

After siRNA transfection, the pathway inhibitor *mnumb* was strongly upregulated by more than 30% in knockdown cells compared to controls (*p* < 0.05, Figure 2A). Meanwhile, Notch pathway elements, including *Notch-1*, *Notch-2* and *hes2* mRNA, were downregulated by more than 50% (*p* < 0.01), more than 30% (*p* < 0.05) and roughly 70% (*p* < 0.05), respectively, relative to control-siRNA transfected cells (Figure 2A). 

### 2.4. Breast Cancer Stem Cell Markers Are Downregulated after MSI Protein Knockdown

The Notch pathway is known to be a key regulator of the BCSC phenotype [24]. Hence, we quantified several stem cell markers to understand the effect of *MSI* knockdown on BCSCs: CD44, a key stemness-associated marker protein [25], was downregulated by more than 20% (*p* < 0.05) in flow cytometric analysis (Figure 2B, representative graph in Figure 2C). *GBX2*, another stem-cell-associated molecule [26], was downregulated 40% compared to non-*MSI*-knockdown controls when quantified by qPCR (*p* < 0.05, Figure 2D). Finally, *vimentin*, a major cytoskeletal element linked to the BCSC phenotype [27], showed lower mRNA expression in MSI-knockdown cells when compared to controls (*p* < 0.01, Figure 2D), a result confirmed using Western blots (Supplementary Figure S3).

### 2.5. Decreased MSI Protein Expression Results in Increased Apoptosis and Reduced Proliferation

Given these previous findings we hypothesized that *MSI* suppression may impact cell survival and cell proliferation.

**Figure 3:**
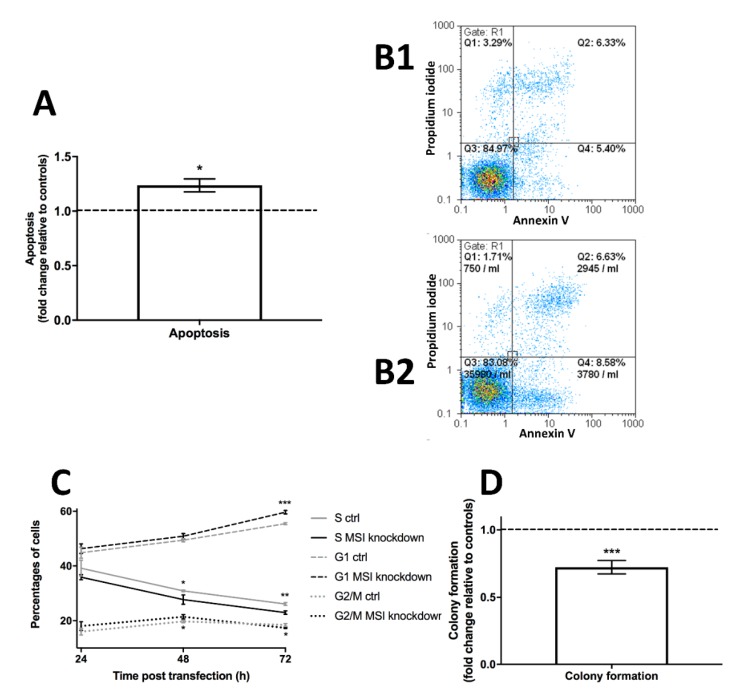
Influence of *Musashi* (*MSI*) RNA-binding protein knockdown on apoptosis, cell cycle progression and colony formation in triple-negative MDA-MB-231 cells. **A**: Apoptosis is upregulated after *MSI* knockdown compared to controls using Annexin V/propidium iodide flow cytometry assay, representative images for control cells in **B1** and for *MSI* knockdown cells in **B2**. Apoptotic cells are shown in Q4. **C**: Changes in cell cycle progression after *MSI* silencing as quantified by DNA staining followed by flow cytometry, compared to respective controls (ctrl). **D**: Colony formation is strongly downregulated after *MSI* knockdown. Cells were transfected with a control siRNA and *MSI-1* and *MSI-2* siRNA, respectively, as detailed in the Methods section (at least *n* = 3, * *p* <0.05, ** *p* < 0.01, *** *p* < 0.001, error bars indicate s.e.m.).

First, we set out to investigate cell apoptosis. During flow cytometry-based measurements, cell apoptosis was upregulated by roughly 25% in *MSI*-knockdown cells compared to controls (*p* < 0.05, Figure 3A, representative graph in Figure 3B). 

Second, we used flow cytometric cell cycle analysis to understand effects on cell proliferation. Measurements at 24, 48 and 72 h post transfection indicated a decline in S phase, no major changes in G2/M phase cells and a relative uptick in G1 phase in *MSI*-deficient cells when compared to controls. Effects were most pronounced after 72 h with significant changes in all phases (G1 phase *p* < 0.001, S phase *p* < 0.01, G2/M phase *p* < 0.05, respectively, Figure 3C).

Finally, building on the two effects detailed before, we used colony formation tests to show that *MSI* knockdown cells were 30% less likely to form colonies compared to controls (*p* < 0.001, Figure 3D). 

### 2.6. Low MSI Expression Leads to Decreased Radioresistance and Reduced Expression of DNA Repair-Related Proteins DNA-PKcs and EGFR

A previous study in glioblastoma demonstrated that *MSI-1* knockdown resulted in lower expression of DNA-Protein Kinase catalytic subunit (DNA-PKcs), a key DNA repair protein, and, subsequently, increased radiosensitivity [21]. However, in other tumor entities, including in breast cancer, radiation effects of MSI proteins have not been investigated. 

**Figure 4:**
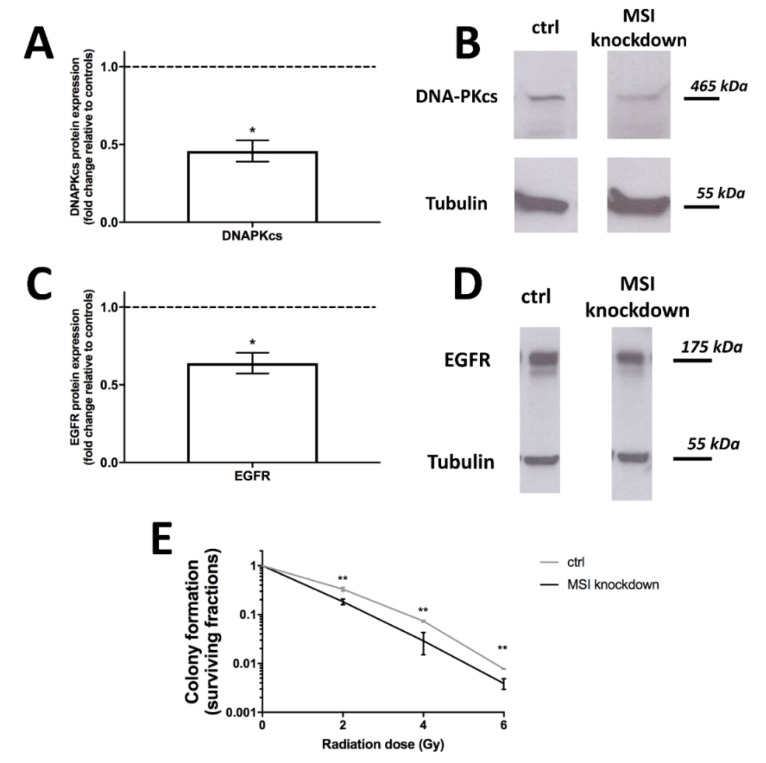
Influence of *Musashi* (*MSI*) RNA-binding protein knockdown on DNA protein kinase catalytic subunit (DNA-PKcs) and Epithelial growth factor receptor (EGFR) protein expression as well as radioresistance in triple-negative MDA-MB-231 cells. **A**: DNA-PKcs expression, as quantified by Western blot, is strongly downregulated after *MSI* knockdown, with representative blots in (**B**). **C**: EGFR expression, as quantified by Western blot, is downregulated after *MSI* silencing, with representative blots in (**D**). **E**: Radioresistance, as quantified by colony formation, is decreased after MSI knockdown and radiation doses of 2, 4 and 6 Gy, respectively, when compared to controls (ctrl). Cells were transfected with a control siRNA and *MSI-1* and *MSI-2* siRNA, respectively, as detailed in the Methods section (at least *n* = 3, * *p* < 0.05, ** *p* < 0.01, error bars indicate s.e.m.).

We thus performed Western blots for DNA-PKcs to evaluate expression in *MSI*-knockdown cells. Protein levels were strongly downregulated (*p* < 0.05, Figure 4A, representative blots in Figure 4B). 

In colon cancer, MSI-1 expression and epithelial growth factor pathway activity were correlated [28], the latter being another key regulator of radioresistance that is specifically targeted in radiation-centered clinical trials [29]. As evidenced by our Western blot results, the epithelial growth factor receptor (EGFR) is downregulated following *MSI* knockdown (*p* < 0.05, Figure 4C, representative blot in Figure 4D).

Finally, the radiation response was also directly investigated. A γH2AX assay to quantify DNA double strand breaks showed no differences between samples and controls (Supplementary Figure S4). However, colony formation was then used to show effects on clonogenic cell survival after irradiation with doses of 2, 4 and 6 Gy. Across all doses, *MSI* knockdown cells were significantly less likely to form colonies (*p* < 0.01 in all cases), thus demonstrating increased radiosensitivity after *MSI* knockdown (Figure 4E).

### 2.7. MSI-Downregulated Cells Exhibit Increased Cell Motility and Invasion

**Figure 5:**
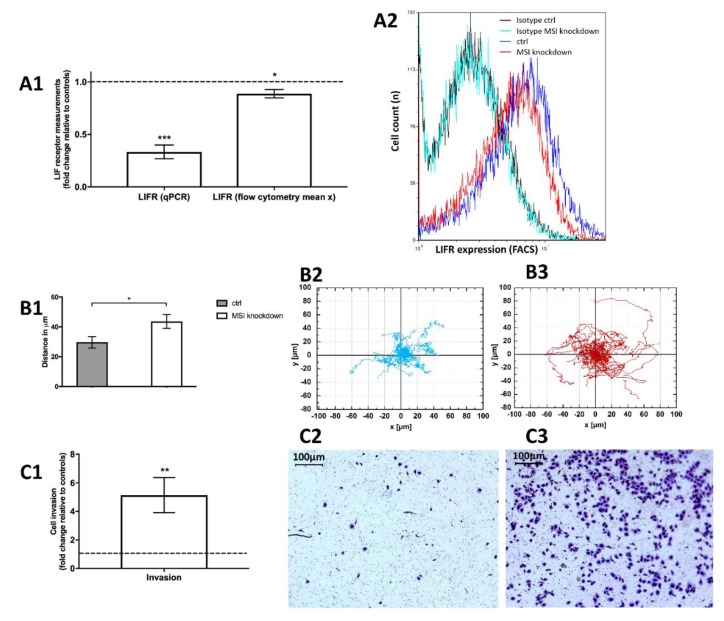
Influence of *Musashi* (*MSI*) RNA-binding protein knockdown on leukemia inhibitory factor receptor (LIFR) expression, cell migration and invasiveness in triple-negative MDA-MB-231 cells. **5A1**: The LIFR is downregulated in *MSI* knockdown cells both in qPCR and in flow cytometric measurements (mean x being the mean fluorescence intensity of the respective sample) with a representative histogram including respective isotypes (unspecific antibodies of the same subclass that show low fluorescence intensity and no discernible difference between samples, thus indicating that changes are due to specific antibody binding) in **5A2** (on a logarithmic x scale). **5B1**: Cell migration distance increases subsequent to *MSI* knockdown compared to controls (ctrl): Cell tracking of control cells (**5B2**) and *MSI* knockdown cells (**5B3**) demonstrates higher motility in the latter. **5C1**: Cell invasiveness is strongly enhanced after *MSI* silencing with representative pictures of invasive cells for controls (**5C2**) and *MSI* knockdown cells (**5C3**). Respective staining was performed with 1% Toluidine blue. Cells were transfected with a control siRNA and *MSI-1* and *MSI-2* siRNA, respectively, as detailed in the Methods section (at least *n* = 3, * *p* < 0.05, ** *p* < 0.01, *** *p* < 0.001, error bars indicate s.e.m.).

Based on a previous study employing Notch inhibitors in endometriosis [30], we could show that the leukemia inhibitory factor receptor (LIFR) expression, a key inhibitor of cell invasion and motility, was downregulated in MSI knockdown cells both in qPCR (by more than 60%, *p* < 0.001, Figure 5A1) and flow cytometry analysis (*p* < 0.05, Figure 5A1,A2).

To understand subsequent effects on cell motility, we performed single cell holographic microscopic investigations. Interestingly, we noted that *MSI* knockdown cells were more mobile than respective controls. We tracked 20 single knockdown and control cells over 30 hours and then analyzed the distance (in μm) from the starting point. Knockdown cells moved significantly further away from the respective starting point compared to controls (*p* < 0.05, Figure 5B). 

We then wanted to understand effects on cell invasiveness. In vitro, *Musashi* knockdown cells were 5 times more likely to migrate through the experimental barrier, the basement membrane-like matrix matrigel (*p* < 0.01, Figure 5C1 with representative findings in Figure 5C2,C3). 

## 3. Discussion

### 3.1. MSI Protein Knockdown Critically Downregulates Stem Cell Characteristics and Cell Cycle Progression While Increasing Apoptosis Subsequent to Notch Pathway Inactivation 

Previous findings indicate that the MSI proteins are likely to enhance Notch signaling pathway activity via targeting and downregulating a pathway inhibitor, m-numb [23]. This holds true in our study as well. Both *MSI-1* and *MSI-2* were well-expressed in triple-negative breast cancer samples and showed strong correlations with Notch pathway elements *Notch-1* and *Notch-2*. *MSI-1* and *MSI-2* also strongly correlated with each other, underlining previously reported homology [9,10]. These findings helped inform the decision to then perform double knockdown experiments.

After knockdown, *mnumb* was expressed higher, while downstream parts of the Notch pathway were repressed. Targeting the Notch pathway has been discussed [31] and may help improve response to cancer therapy [32]. We show that both *Notch-1* and *Notch-2* are upregulated in cancer samples compared to healthy tissue, thus emphasizing the important role and dysregulation of the Notch pathway in breast cancer.

As the Notch pathway exerts a strong influence over BCSCs [24], we aimed to investigate several stem cell characteristics not previously reported as targeted by MSI silencing in breast cancer:

- We show that CD44 is downregulated via *MSI* knockdown in breast cancer. CD44 is a key stem cell marker in mammary malignancies and it has been shown that as few as 100 CD44^(high)^ cells may promote tumorigenesis in breast cancer [25]. Our findings are in line with studies in colon cancer indicating a positive relationship between MSI proteins and CD44 [33,34].
- *GBX2*, a key marker for stem cell progenitors [26], is also downregulated after MSI knockdown. Its downregulation is known to inhibit proliferation [26], prompting further proliferation analyses (see below).
- The mesenchymal protein vimentin has been described as a stem cell regulator in mouse models that leads to reduced regenerative capacity and is associated with tumor sphere formation [27].

All three cancer stem cell markers strongly pointed to effects on proliferation and, potentially, apoptosis, given that stem cells are known to be resistant to cell death [35]. Both investigations showed significant results:

- The cell cycle analysis findings were fairly similar to previous quantifications published by our group in endometrial carcinoma [14]. In sum, we see a strong increase in G1 phase cells at the expense of S and no substantial change in G2/M phase cell proportions after 72 hours, suggesting an anti-proliferative effect. This effect seems to slowly develop between 24 and 72 hours with its peak after 72 hours.
- The apoptosis assay demonstrated that the antiproliferative effect also seems to carry pro-apoptotic properties: Knockdown cells were significantly more likely to bind annexin V, indicating apoptotic features. Again, this is well in line with a previous study in endometrial cancer [14].

Both experiments clearly suggested reduced colony formation after *MSI* knockdown, a finding which we could subsequently demonstrate. Hence, we show that *MSI* knockdown uniformly decreases proliferation and colony formation while increasing apoptosis.

### 3.2. MSI Protein Knockdown Reduces Breast Cancer Radioresistance via Downregulation of EGFR and DNA-PKcs

Musashi proteins have only recently come into focus as regulators of radioresistance with studies in glioblastoma [20,21] and colon cancer [22]. However, investigations in other tumor entities, including in breast cancer, have not been performed. 

Our colony formation experiments post radiation demonstrate a strong decrease in proliferative capacity at all radiation doses of 2, 4 and 6 Gy. Given this comes on top of the previously discussed antiproliferative effect in unirradiated cells, this indicates a dramatic decline in colony formation overall, both without and (even more so) with radiation. 

Subsequently, we aimed to understand the underlying mechanisms facilitating the documented radiosensitization. We believe the causes are multifactorial:

- First and most importantly, the Notch pathway has been described to confer radioresistance through enhancing cancer stem cell properties [36,37]. Conversely, Notch signaling is upregulated after irradiation [38]. We have demonstrated a correlation between MSI expression and Notch elements in primary breast tissues as well as a downregulation of Notch signaling after MSI knockdown. Decreased Notch activity may thus confer reduced radioresistant properties.
- CD44, one of aforementioned cancer stem cell markers, is closely linked to radioresistance, e.g., in bladder [39] and pancreatic cancer [40]. CD44 was downregulated subsequent to MSI knockdown in our study.
- Epithelial mesenchymal transition (EMT) has also been linked to radioresistance [41]. Vimentin is key to EMT [42]. With vimentin strongly reduced after MSI knockdown, this is another possible stem-cell-based explanation for the loss of radioresistance.
- In glioblastoma, de Araujo and colleagues demonstrated a decrease in DNA-PKcs expression [21]. In our study, we demonstrate the same effect for breast cancer. A decrease in this key DNA repair protein is known to sensitize breast cancer to radiation [43] and the protein has been suggested as a therapeutic target [44]. Interestingly, the decrease in DNA-PKcs may also explain why we were unable to see changes in double strand breaks via γH2AX assay. The γH2AX assay does not directly measure double strand breaks, but rather the (related) γ-phosphorylation of histones. However, this process is mediated by DNA-PKcs as aptly summarized by An et al.: “DNA-PKcs plays a dominant role in the regulation of H2AX phosphorylation in response to DNA damage and cell cycle progression” [45]. Thus, with potentially more DNA damage, but less DNA-PKcs to indicate damage via histone phosphorylation, we believe it is ultimately understandable why no overall change was seen in the γH2AX assay.
- Lastly, EGFR is also well known for its mediation of radioresistant properties with new clinical trials to target its effect underway [29,46]. *MSI* knockdown downregulates EGFR, thus providing another potential mechanism to enhance radiosensitivity. EGFR also plays a key role for BCSC activity [47], demonstrating yet again that many of the aforementioned mechanisms are closely related.

Our experiments clearly suggest a decrease in radioresistance subsequent to *MSI* knockdown with several, largely intertwined underlying mechanisms most likely responsible.

### 3.3. MSI Protein Knockdown Results in a Higher Cell Invasiveness and More Migration In Vivo, Possibly due to Downregulation of the LIF Receptor

Based on previous findings in endometriosis linking MSI proteins to LIFR expression [30] we aimed to investigate this interplay in breast cancer. Similar to endometriosis, the LIFR was downregulated in breast cancer. However, in breast cancer, the LIFR is known as a metastasis suppressor [48] given its role as an upstream part of the Hippo-YAP pathway: High LIFR expression suppresses metastases by inactivating the transcriptional coactivator YES-associated protein (YAP) through a cascade of multiple phosphorylation processes. Conversely, low LIFR expression induces invasion and enhances cell migration through activation of YAP. Thus, LIFR is known to be inversely correlated with metastasis formation [48]. This antitumorigenic role is underlined by the fact that LIFR is less expressed in breast cancer tissues compared to normal breast tissues [49]. LIFR overexpression may also confer dormancy in breast cancer metastases to the bone [50]. Finally, high LIFR expression in breast cancer has also been correlated with better overall survival (OS) [48].

With *MSI* knockdown leading to LIFR downregulation, a pro-metastatic, pro-migration effect seemed plausible which led us to perform invasion and migration assays. Cell migration was upregulated in breast cancer cells subsequent to *MSI* knockdown, with cells traveling longer distances. Invasion assays indicated highly enhanced invasive capacities in cells subsequent to *MSI* silencing. 

Comprehensively, our results help identify important properties of Musashi RNA-binding proteins. We show that *MSI* silencing enhances pro-apoptotic, anti-proliferative and anti-radioresistant signaling. Furthermore, stem cell markers are downregulated. In sum, this indicates a potentially high therapeutic value of targeting MSI RNA-binding proteins. However, upregulated cell migration and invasiveness clearly constitute troublesome consequences of MSI silencing. For *MSI* knockdown to be therapeutically valuable in breast cancer, it is vital to further elucidate and potentially counteract pro-invasive and pro-migration properties conferred by *MSI* silencing given that metastatic capability is a key deciding factor for OS.

This study has three key limitations: First, the majority of experiments were conducted in vitro, not in vivo. Second, only one of the Musashi proteins and one stem cell marker were quantified by Western blot, while most of the remaining gene expression was quantified via qPCR only. Western blots for all gene expression experiments would have provided a higher level of evidence. However, qPCR results are in line with provided Western blot results of the two proteins and previous literature. Third, a triple-negative cell line was chosen and only TNBC primary samples were investigated, limiting applicability, especially for hormone receptor positive breast cancer where additional studies are needed. Nonetheless, this study is the first to offer major insights into some consequences of *MSI* knockdown in breast cancer, including demonstrating effects on some stem cell characteristics, cell cycle progression, apoptosis, radiotherapy and cell invasiveness and migration. 

In conclusion, in our study, we demonstrate that Musashi protein knockdown downregulates several important stem cell characteristics in breast cancer, including CD44, vimentin and GBX2, likely due to Notch pathway downregulation. Subsequently, cell cycle progression is altered, proliferation repressed and apoptosis upregulated. After *MSI* knockdown, breast cancer radioresistance is decreased due to fewer stem cell characteristics, lower Notch signaling, as well as DNA-PKcs and EGFR downregulation. However, with the LIF receptor downregulated, *MSI* silencing also leads to enhanced invasiveness and migration, severely impacting its therapeutic potential.

## 4. Materials and Methods 

### 4.1. Cell Line and Transfection

The triple-negative breast cancer cell line MDA-MB-231 was acquired from American Type Culture Collection (ATCC)/LGC Standards (Wesel, Germany). The cell line was authenticated via short tandem repeat (STR) analysis. Cells were cultured as previously described [51]. Transient *MSI* knockdown was performed via transfection of respective *MSI-1* and *MSI-2* siRNAs (Supplementary Table S1). Given 90% homology [10], both MSI proteins were knocked down. Success of transfection was investigated via quantitative polymerase chain reaction (qPCR) analysis. 

### 4.2. qPCR

mRNA was isolated 48 h after siRNA transfection using the RNeasy Mini Kit (Qiagen, Venlo, The Netherlands). For reverse transcription, the High-Capacity cDNA Reverse Transcription Kit was used, and qPCR was then performed on a Rotor-Gene Q machine (Qiagen). All proceedings were handled according to the manufacturer’s instructions and as previously described [7]. A list of the primers used can be found in Supplementary Table S2.

### 4.3. Western Blot Analysis

1 × 10^7^ cells were trypsinized and washed with PBS 48 h after transfection. The final pellet was resuspended and incubated for 30 min on ice. After centrifugation at 13,000 rpm for 10 min, gel electrophoresis and Western blotting were performed as previously described [52]. In detail, a precast gradient gel of 4% to 20% (Bio-Rad Laboratories, Feldkirchen, Germany) was used for electrophoresis and 30 µg of proteins were loaded. Prestained standards were used for molecular weight estimation. Protein separation was performed at 25 mA constant current. Protein transfer to nitrocellulose membrane was done overnight at 10 V constant voltage. After blocking of free binding sites by treating nitrocellulose membrane for 1 h with 5% skim milk in 0.1% TBS-Tween (TBST), incubation with the primary antibody followed overnight at 4 °C with antibody dilutions as recommended by the manufacturer. After washing with TBST three times, secondary horseradish peroxidase-conjugated antibodies diluted in blocking buffer were applied for 1 h. The membranes were washed three times with TBST and antibody binding was visualized using peroxide substrate (Thermo Fisher Scientific, Waltham, MA, USA) with a Fusion SL System (Peqlab, Erlangen, Germany) employed for quantification. Antibody details are shown in Supplementary Table S3. Chemiluminescence was detected on films and intensity was analyzed by ImageJ software (NIH, Bethesda, MD, USA) for PC. Tubulin was used as endogenous control protein and detected and quantified after stripping the former used membranes with a buffer containing 0.87% NaCl and 0.75% glycine at pH 2.5. Afterward, the membranes were washed and re-incubated with mouse anti-human tubulin (Sigma, Deisenhofen, Germany) followed by the subsequent detection procedure as described previously. 

### 4.4. Flow Cytometry

CD44 and LIFR positivity were analyzed 48 h after transfection using CD44 APC (BD Pharmingen, Franklin Lakes, NJ, USA) or phycoerythrin (PE) conjugated mouse anti-human LIFR antibodies (R&D Systems, Minneapolis, MN, USA) and their corresponding isotype controls (APC isotype from BD Pharmingen, PE isotype from R&D Systems). All experiments were performed according to manufacturer’s instructions and as previously described [7]. Catalogue numbers for the antibodies used are given in Supplementary Table S4.

### 4.5. Cell Colony Formation

First, cells were transfected as described above. Then, 24 h after transfection, cells were irradiated with doses of 2, 4 and 6 Gy, respectively. Afterwards, cell culture dishes (Nunc, Langenselbold, Germany) were used and predefined numbers of cells were seeded and then incubated for 10 days. A colony was defined as a contiguous cell group of more than 50 cells. Plating efficiency (PEf) was determined as PEf = colony number/number of seeded cells. Surviving fractions (SFs) of radiated cells were calculated relative to non-irradiated controls (SF = PEf(irradiated) / PEf(control)). For irradiation, a TrueBeam linear accelerator (Varian, Palo Alto, CA, USA) was used. 

### 4.6. Cell Cycle Progression

Cells were seeded in 6-well-plates and transfected after 24 h. Another 24 h later, transfection medium was changed back to cell line-specific medium. 24, 48 and 72 h after this, cell cycle status was analyzed via flow cytometry. For this, cellular DNA was stained with 4’,6-diamidino-2-phenylindole DAPI (Cystain, Sysmex/Partec, Görlitz, Germany) and fluorescence intensity was measured as previously described [14]. Cell cycle distribution was calculated using FloMax software (Quantum Analysis, Münster, Germany).

### 4.7. γH2AX

Cells were seeded in 6-well-plates and, 24 h later, transfected as detailed above. 24 h after transfection, cells were irradiated with 2 Gy using a clinical TrueBeam linear accelerator (Varian, Palo Alto, CA, USA) with one control-transfected and one MSI-siRNA-transfected sample going unirradiated. Afterwards, cells were fixated in 70% (*v*/*v*) ice cold ethanol after pre-defined periods of time (1 h, 2 h, 4 h, 6 h, 24 h) and stored in a freezer until measurement. Staining procedure and flow cytometric analysis was done as previously described [52] with the exception that a FITC labeled anti-phospho-histone H2AX antibody (Ser139) was used (clone JBW301, catalogue number 05-636, Merck Millipore, Darmstadt, Germany).

### 4.8. Apoptosis

Again, 24 h after transfection, medium was exchanged as detailed above, with the apoptosis assay initiated 24 h after that. After washing with phosphate-buffered serum (PBS), cells were treated using the Annexin V/propidium iodide (PI) assay (formerly Invitrogen, now Thermo Fisher Scientific) as previously described [53]. 

### 4.9. Invasion

Invasion was measured via Matrigel invasion assay. As usual, 24 h after transfection, the medium was exchanged and another 24 h passed before invasion assay was initiated. For this, 25,000 cells were seeded on the Matrigel-coated 8.0 µm PET membranes of Corning® BioCoat^TM^ Matrigel® Invasion Chambers (Corning, New York, NY, USA) with 10% Fetal Calf Serum (FCS) medium and incubated for 24 h. Afterwards, 10% FCS culture medium was given to the lower cell-free part of the invasion chamber while the upper-compartment medium was exchanged to contain no FCS, thus generating a chemotactic gradient. Then, 24 hours later, cells in the lower compartment were fixed, stained with 1% Toluidine Blue (Sigma-Aldrich, St. Louis, MO, USA) and counted, as described previously [54].

### 4.10. Digital Holographic Microscopy

Digital holographic microscopy [55] was used to determine cell migration behavior. Experiments were conducted as previously described [56]. A total of 250,000 cells were transiently transfected with respective siRNAs and seeded into petri dishes (µ-Dish with glass lid, Ibidi, Gräfeling, Germany). After 24 hours, the transfection medium was changed to DMEM + 10% FCS + 20 mM HEPES. Starting from this timepoint, digital holograms were recorded every 15 min for 30 h from which, subsequently, quantitative phase images were reconstructed. A previously described software was used for cell tracking [57]. To quantify cell motility, the maximum cell distance from the respective starting point was recorded after 30 h and compared between MSI knockdown and control cells.

### 4.11. Primary Tissue

This study was approved by the Institutional Review Board of the National Cancer Institute (NCI)-Egypt (IRB#00004025). All patients signed a consent form to participate in the investigation. Besides primary tissue, patient characteristics were also collected. One part of carcinoma tissues was fixed in 10% PBS-buffered formalin and the other part was used to isolate total RNA using Invitrogen™ RNAqueous total RNA isolation kit (Thermo Fisher Scientific). The isolated RNA was reverse transcribed into cDNA using the high capacity cDNA Kit (Thermo Fisher Scientific). Relative gene expression was assessed using SYBR Green master mix in StepOnePlus detection System (Applied Biosystems, San Francisco, CA, USA). Relative gene expression was evaluated using the 2^−∆∆Ct^ method after normalization to the house keeping gene RPLO (Qiagen) and data were represented as log2-transformed fold change. For comparison, 5 healthy, non-cancerous breast tissues obtained during reduction mammoplasty were used.

### 4.12. Statistical Analysis

Experiments were performed at least three times in duplicates. Data were tested for differences using Student’s t-test with the level of significance defined as *p* < 0.05. Fold changes are shown as mean ± standard error of the mean (s.e.m.) if not otherwise stated.

For the primary breast cancer samples, Spearman’s rank correlation was used to compare mRNA expressions, again with the level of significance defined as *p* < 0.05. For expression comparisons between breast cancer samples and healthy tissue samples, the Mann-Whitney U test with the same threshold of significance was used.

## Figures and Tables

**Table 1:** Patient characteristics. N = number, SD = standard deviation.

Characteristic	N (%)
Age (years)	
Range	34–63
Mean ± SD	52 ± 8
Menopausal status	
Premenopausal	8 (42.1%)
Postmenopausal	11 (57.9%)
Tumor size (cm)	
T1	3 (15.8%)
T2	9 (47.4%)
T3	5 (26.3%)
T4	2 (10.5%)
Tumor grade	
G I	1 (5.3%)
G II	17 (89.4%)
G III	1 (5.3%)
Positive axillary lymph nodes	
N0	2 (10.5%)
N1	9 (47.4%)
N2	5 (26.3%)
N3	3 (15.8%)
Lymphovascular invasion	
Negative	11 (57.9%)
Positive	8 (42.1%)

## Supplementary Materials

Supplementary materials can be found at https://www.mdpi.com/1422-0067/21/6/2169/s1.

## Abbreviations

ALDH	Aldehyde dehydrogenase
BCSC	Breast cancer stem cell
Ctrl	Control
DAPI	4’,6-diamidino-2-phenylindole
DNA-PKcs	DNA protein kinase catalytic subunit
EGFR	Epithelial growth factor receptor
EMT	Epithelial mesenchymal transition
FACS	Fluorescence-activated cell sorting
FCS	Fetal Calf Serum
LIFR	Leukemia inhibitory factor receptor
MDR	Multidrug resistance
Mnumb	Mammalian numb
MSI	Musashi (RNA-binding proteins)
MSI-1	Musashi RNA-binding protein 1
MSI-2	Musashi RNA-binding protein 2
OS	Overall survival
PE	Phycoerythrin
PEf	Plating efficacy
PI	Propidium iodide
qPCR	Quantitative polymerase chain reaction
s.e.m.	Standard error of the mean
SF	Surviving fraction
siRNA	Small interfering RNA
STR	Short tandem repeat
TBST	TBS-Tween
YAP	YES-associated protein
