# Supplementary material for: Knockdown of Musashi RNA Binding Proteins Decreases Radioresistance but Enhances Cell Motility and Invasion in Triple-Negative Breast Cancer
Source: Int J Mol Sci. 2020 Mar 21;21(6):2169. doi: 10.3390/ijms21062169 (PMC7139790; doi:10.3390/ijms21062169)
Supplement: Supplementary file 1 [file ijms-21-02169-s001.pdf]

Supplementary Materials

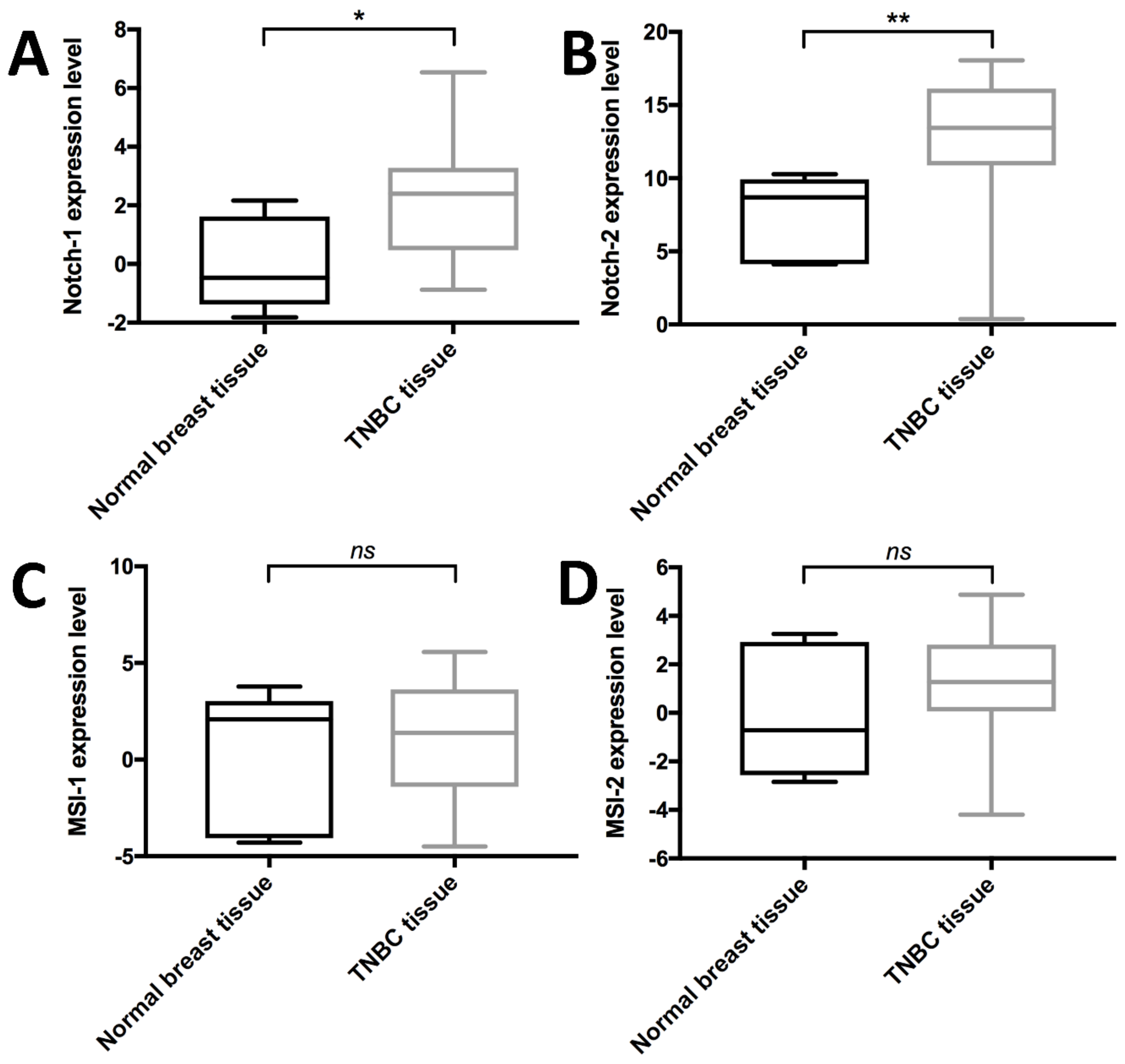

Supplementary figure S1. Differences in expression between normal breast tissue samples (n = 5) and triple-negative breast cancer (TNBC) samples (n = 19) were assessed via quantitative polymerase chain reaction (qPCR). After normalization to the housekeeping gene, respective gene expression was evaluated using the $2^{-\Delta\Delta Ct}$ method with data then presented as log2-transformed fold change. Mann Whitney U test was used for correlation (* $p < 0.05$, ** $p < 0.01$, boxplot indicates interquartile range). **S1A**: *Notch-1* expression is higher in TNBC tissues compared to normal breast tissues ($p < 0.05$). **S1B**: *Notch-2* expression is higher in TNBC tissues compared to normal breast tissues ($p < 0.01$). **S1C**: *MSI-1* expression is not significantly different in TNBC tissues when compared to normal breast tissues ($p > 0.05$). **S1D**: *MSI-2* expression is not significantly different in TNBC tissues when compared to normal breast tissues ($p > 0.05$).

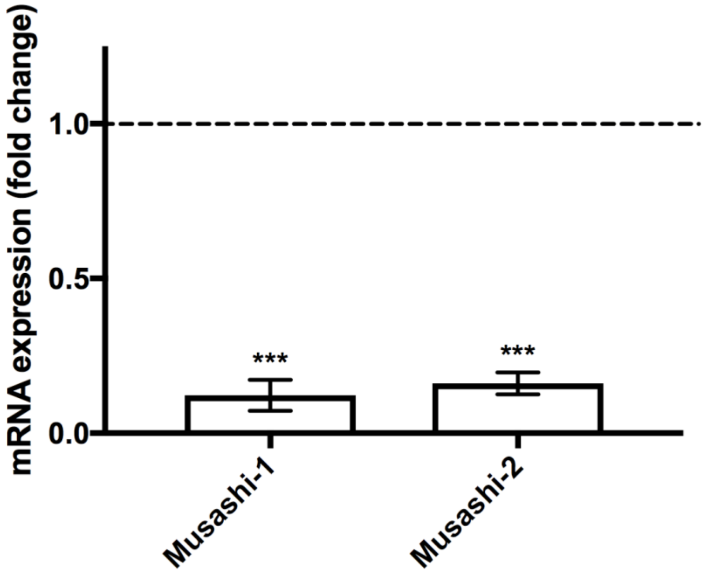

Supplementary Figure S2. *Musashi-1* (*MSI-1*) and *Musashi-2* (*MSI-2*) levels subsequent to knockdown with *MSI-1* & *MSI-2* siRNA, as quantified by quantitative polymerase chain reaction (qPCR). Both mRNAs were repressed by roughly 80%. Cells were transfected with a control siRNA and *MSI-1* & *MSI-2* siRNA, respectively, as detailed in the methods section (at least $n = 3$, *** $p < 0.001$, error bars indicate standard error of the mean (s.e.m.)).

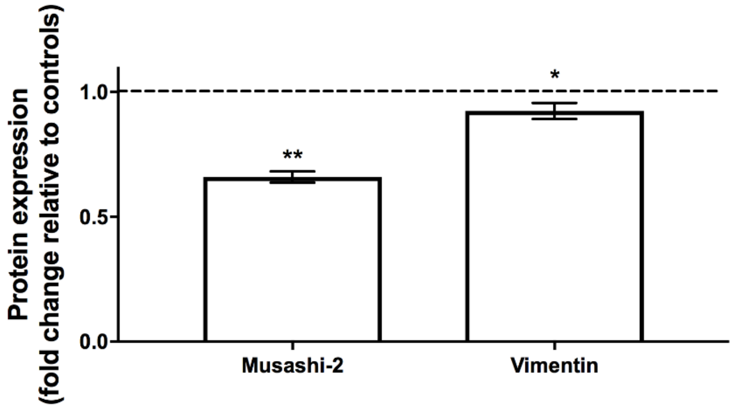

Supplementary Figure S3: Changes in MSI-2 and Vimentin protein levels subsequent to knockdown with *MSI-1* & *MSI-2* siRNA, as quantified by western blot. Cells were transfected with a control siRNA and *MSI-1* & *MSI-2* siRNA, respectively, as detailed in the methods section (at least $n = 3$, * $p < 0.05$, ** $p < 0.01$, error bars indicate standard error of the mean (s.e.m.))

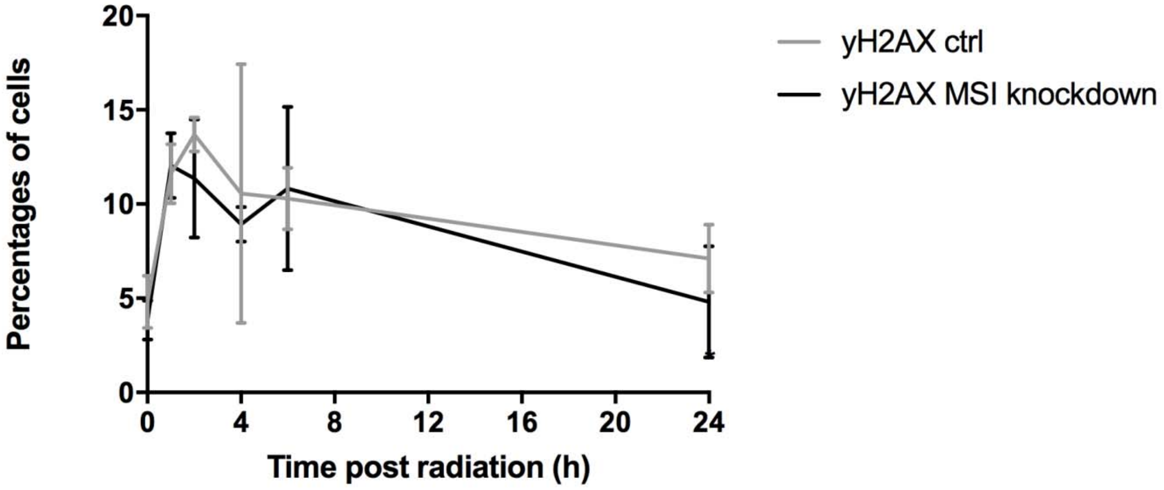

Supplementary Figure S4. γH2AX measurements in *Musashi* (*MSI*) knockdown cells and respective controls. No significant differences were observed at all timepoints. Cells were transfected with a control siRNA and *MSI-1* & *MSI-2* siRNA, respectively, as detailed in the methods section.

Supplementary Table S1. siRNAs used for experiments.

siRNA	Manufacturer	Manufacturer ID	UniGene ID
MSI-1	Thermo Fisher Scientific	S8980	Hs.158311
MSI-2	Thermo Fisher Scientific	S42757	Hs.658922
Negative control	Thermo Fisher Scientific	4390843	-

Supplementary Table S2. TaqMan primers used for qPCR experiments.

Gene	Manufacturer	Primer Sequence
mnumb	Thermo Fisher Scientific hs00377772-M1	*Full product details can be found on the manufacturer's website using the catalogue number.*
Notch-1	Thermo Fisher Scientific hs00413187_M1	
Notch-2	Thermo Fisher Scientific hs01050719_M1	
hes2	Thermo Fisher Scientific hs00219505_M1	
GBX2	Thermo Fisher Scientific hs00230965_M1	
LIFR	Thermo Fisher Scientific hs01123581_M1	
vimentin	Biolegio (Nijmegen, The Netherlands)	Fwd: TCAGCATCACGATGACCTTGAA<br>Rev: CTGCAGAAAGGCACTTGAAAGC
GAPDH	Biolegio	Fwd: CCCAGCAAGAGCACAAGAGG<br>Rev: GGTCTACATGGCAACTGTGAGGA

Supplementary Table S3. Antibodies used for western blotting.

Target	Manufacturer	Catalogue number
MSI-2	Santa Cruz	SC-83160
Vimentin	Santa Cruz	SC-32322
DNA-PKcs	R&D-Systems	AF3415
EGFR	Abcam (Cambridge, United Kingdom)	AB32430
Tubulin	Sigma-Aldrich	T5168
Rabbit Anti-Goat IgG Peroxydase	Sigma-Aldrich	401515
Goat-Anti-Rabbit IgG Peroxidase	Sigma-Aldrich	401353
Goat-Anti-Mouse IgG HRP	Sigma-Aldrich	12-349

Supplementary Table S4. Antibodies used for flow cytometry.

Target	Manufacturer	Catalogue number
CD44	BD Biosciences	559942
LIFR	R&D-Systems	FAB249A
APC isotype	BD Biosciences	555576
PE isotype	R&D-Systems	IC002A
